# Enhanced Osteogenesis Potential of MG-63 Cells through Sustained Delivery of VEGF via Liposomal Hydrogel

**DOI:** 10.3390/gels9070562

**Published:** 2023-07-10

**Authors:** Milton Hongli Tsai, Rohaya Megat Abdul Wahab, Shahrul Hisham Zainal Ariffin, Fazren Azmi, Farinawati Yazid

**Affiliations:** 1 Discipline of Orthodontics, Department of Family Oral Health, Faculty of Dentistry, Universiti Kebangsaan Malaysia, Kuala Lumpur 50300, Malaysia; p106712@siswa.ukm.edu.my (M.H.T.); rohaya_megat@ukm.edu.my (R.M.A.W.); 2 Department of Biological Sciences and Biotechnology, Faculty of Science and Technology, Universiti Kebangsaan Malaysia, Bangi 43600, Malaysia; hisham@ukm.edu.my; 3 Faculty of Pharmacy, Universiti Kebangsaan Malaysia, Kuala Lumpur 50300, Malaysia; fazren.azmi@ukm.edu.my; 4 Discipline of Pediatric Dentistry, Department of Family Oral Health, Faculty of Dentistry, Universiti Kebangsaan Malaysia, Kuala Lumpur 50300, Malaysia

**Keywords:** hydrogel, liposomes, MG-63 osteoblast-like cells, osteogenesis, vascular endothelial growth factor

## Abstract

The challenges of using VEGF to promote osteoblastic differentiation include a short half-life and a narrow therapeutic window. A carrier system combining hydrogel and liposomes may improve the therapeutic efficacy of VEGF for bone regeneration. This study aimed to investigate the effects of delivery of VEGF via liposomal hydrogel on the osteogenesis of MG-63 cells. Liposomal hydrogel scaffold was fabricated and then characterized in terms of the morphological and chemical properties using FESEM and FTIR. In 2.5D analysis, the MG-63 cells were cultured on liposomal hydrogel + VEGF as the test group. The osteogenic effects of VEGF were compared with the control groups, i.e., hydrogel without liposomes + VEGF, osteogenic medium (OM) supplemented with a bolus of VEGF, and OM without VEGF. Cell morphology, viability, and differentiation and mineralization potential were investigated using FESEM, MTT assay, ALP activity, and Alizarin red staining. The characterization of scaffold showed no significant differences in the morphological and chemical properties between hydrogel with and without liposomes (*p* > 0.05). The final 2.5D culture demonstrated that cell proliferation, differentiation, and mineralization were significantly enhanced in the liposomal hydrogel + VEGF group compared with the control groups (*p* < 0.05). In conclusion, liposomal hydrogel can be used to deliver VEGF in a sustained manner in order to enhance the osteogenesis of MG-63 cells.

## 1. Introduction

The treatment of critical-sized bone defects (CSD) due to traumatic injuries, tumor resection, or congenital diseases has always been a challenge in the field of medicine and dentistry because spontaneous healing cannot occur in the absence of bone grafts [1]. One common example of a congenital bony defect in the orofacial region is the cleft alveolar and palate, whereby the autograft harvested from the patient’s anterior iliac crest is the most reliable graft material used for closing the bone defect at the alveolar cleft, with success rates reported to be as high as 96.2% [2]. Till today, autogenous bone grafts are still considered the “gold standard”, because they fulfil all the ideal requirements for bone regeneration by having osteogenic, osteoconductive, and osteoinductive properties [3,4]. However, such autograft is often associated with significant donor-site morbidity and psychological complications, which are quite a lot to endure for school-aged cleft patients [5]. Non-autogenous bone grafts such as allografts and xenografts are osteoconductive and mildly osteoinductive, but they lack the osteogenic capacity of autografts and they may carry the risk of infectivity and immune rejection [6]. To address these issues, bone tissue engineering (BTE) has developed synthetic bone substitutes as promising strategies in regenerative therapies. This type of bone substitute generally consists of natural, synthetic, or composite scaffolds, modified with osteoprogenitor cells and/or incorporated with growth factors [7,8]. Hence, the “triad” of osteogenic cells, osteoconductive scaffold materials, and osteoinductive signals (growth factors) successfully recapitulates the properties of autograft, omitting the need for invasive donor-site surgeries [9].

Nevertheless, BTE is such a complex subject of research that even though countless studies have been carried out in the field of BTE, there are still many critical variables yet to be optimized and standardized through in vitro, and subsequently in vivo, animal studies before BTE can be used safely and effectively in clinical applications [10]. One such parameter that requires further fine-tuning is the efficacy of osteogenic differentiation. Thus, it is essential to investigate the choice of signaling factors that may induce a more efficient cellular expansion and osteogenic differentiation by manipulating the culture microenvironment [11]. 

Among the different types of mediators in osteogenesis, vascular endothelial growth factor (VEGF) is an interesting growth factor that is capable of coupling effects of angiogenesis and osteogenesis during both bone formation and regeneration. In this coupling mechanism, VEGF performs a twofold function: inducing angiogenesis by acting on endothelial cells, which is crucial for the recruitment of osteoprogenitor cells, and promoting osteogenesis by direct action on non-endothelial cells (osteoblasts and osteoclasts) that express VEGF receptors [12,13]. Nevertheless, the use of VEGF is yet to be implemented in bone regenerative therapies in a clinical setting. Some of the challenges are the relatively short half-life and narrow therapeutic window that limit the therapeutic efficacy of VEGF for bone regeneration [14]. In other words, it is very difficult to sustain the bioavailability of the growth factor without exceeding the toxicity threshold.

The solution to this problem lies in the selection of a carrier system to improve the release kinetics of the growth factor (VEGF). Hydrogels, a unique type of extracellular matrix-like scaffold with excellent biocompatibility, can allow complete encapsulation of cells and growth factors and facilitate cell expansion and differentiation. Some of the promising hydrogel systems are chitosan, alginate, gellan gum, and hyaluronan [15,16,17]. Among them, thermosensitive hydrogel is produced via physical gelation method, utilizing chitosan, which is a natural polymer derived from the shells of shellfish, an abundant waste product of the seafood industry (renewable resource). Thus, the fabrication of liposomal hydrogel is economical and environment friendly without the need for complex chemical stimuli and expensive equipment [18]. Chitosan has a good biocompatibility property and forms non-toxic oligosaccharide on degradation [19]. Its thermos-responsive nature allows the hydrogel to reach the target site with minimum invasiveness to deliver preloaded cells or biomolecules and then undergo gelation in situ [20]. In addition, nanoliposomes can function as secondary carriers to further minimize the burst release effect when hydrogel is used as the sole carrier. Therefore, a potential solution is to combine both liposomes and chitosan hydrogel, making use of the advantages of both materials. This type of hybrid hydrogel has already been used for other purposes in the medical field, particularly for drug delivery [21]. Hence, the aim of the current study was to investigate the application of liposomal hydrogel as a promising strategy yet to be extended to the field of BTE.

## 2. Results

### 2.1. Characterization of Scaffold

#### 2.1.1. Characterization of VEGF-Loaded Liposomes

The particle sizes, PDI, and zeta-potentials of the liposomes measured by the dynamic light scattering (DLS) method are summarized in Table 1. Liposomes loaded with 100 ng/mL VEGF were fabricated with an average size of 171 nm, an average polydispersity index (PDI) of 0.174, and zeta-potential of +38.5 mV. There were no significant differences in size, PDI, or zeta-potential between liposomes with and without VEGF (*p* > 0.05). 

**Figure 1:**
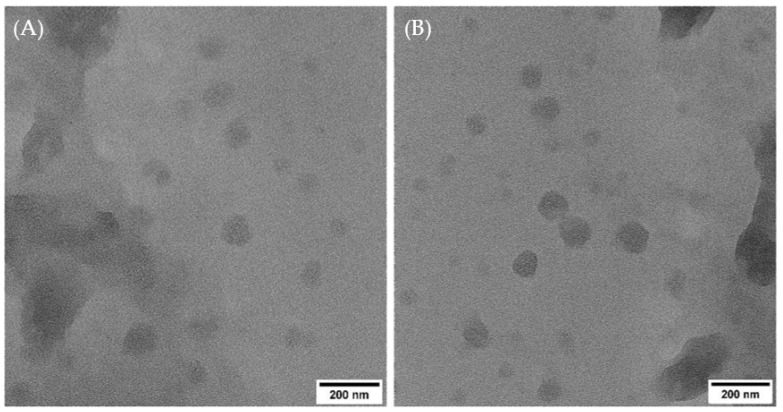
TEM images of (**A**) liposomes without VEGF and (**B**) liposomes with VEGF. (Scale bar: 200 nm.)

The morphology of the liposomes with and without VEGF under transmission electron microscope (TEM) is shown in Figure 1. The particle sizes correlated well with measurements obtained from the nanosizer. The liposomes demonstrated spherical unilamellar morphology and dispersed nature.

#### 2.1.2. Microstructure and Porosity of Hydrogels

**Figure 2:**
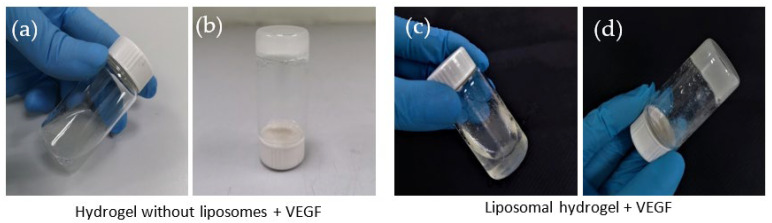
Photographs of the process of gelation for hydrogel without liposomes (**a**,**b**) and liposomal hydrogel (**c**,**d**). The tube inversion method was used to determine the gelation time (**b**,**d**).

The gelation time for hydrogel without liposomes was 10.5 ± 0.52 min but only 7.21 ± 0.43 min for liposomal hydrogel according to the test tube inverting method. The difference was statistically significant (*p* < 0.05). The transparent hydrogel solutions at 4 °C were transformed into non-transparent semisolid hydrogels (Figure 2a–d). 

**Figure 3:**
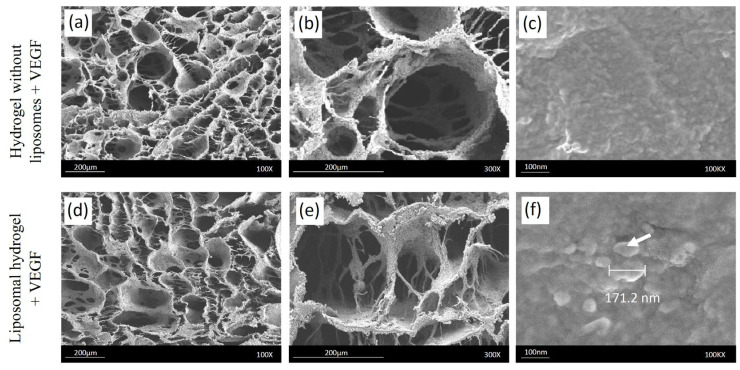
Field emission scanning electron microscope images of hydrogel without liposomes (**a**–**c**) and liposomal hydrogel (**d**–**f**) at 100×, 300×, and 100k× magnifications. The white arrow (**f**) indicates the presence of liposomes in the liposomal hydrogel.

After gelation, the hydrogel without liposomes and liposomal hydrogel were freeze-dried and observed under FESEM. The hydrogels demonstrated a porous and sponge-like microstructure (Figure 3a–f). Successful incorporation of the liposomes in the hydrogel could be verified through Figure 3f, the size of which agreed with the findings from the nanosizer. 

The average pore size and porosity are presented in Table 2. The average pore diameters of hydrogel without liposomes and liposomal hydrogel were 201.4 ± 24.5 µm and 205.8 ± 25.5 µm, respectively. The porosity of the hydrogel without liposomes was 81.1 ± 3.3%, while the porosity of the liposomal hydrogel was 84.0 ± 2.9%. There were no significant differences in the pore size and porosity of hydrogels with or without liposomes.

#### 2.1.3. Chemical Properties of Hydrogels Using FTIR Spectroscopy 

**Figure 4:**
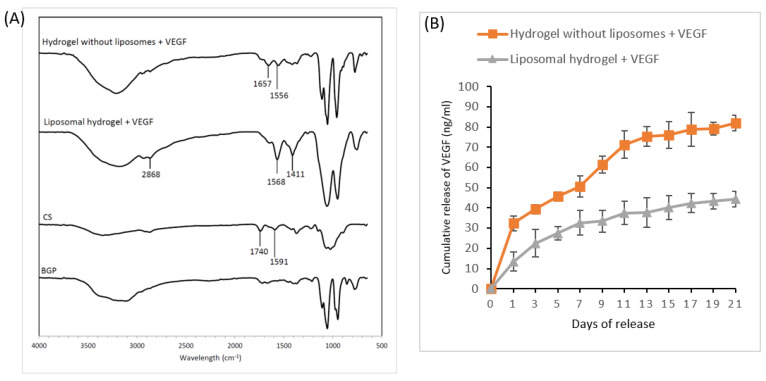
(**A**) The FTIR spectra of hydrogel without liposomes and liposomal hydrogel: (**B**) The cumulative release profile of VEGF from hydrogel with and without liposomes over 21 days. The cumulative release of VEGF in ng/mL was determined as a function of time by VEGF ELISA kit at 450 nm. Each value is expressed as mean ± SD (*n* = 3 per group). Chitosan, CS; β-glycerophosphate, BGP.

The FTIR spectra are displayed in Figure 4A. In general, the spectra of the hydrogel without liposomes and the liposomal hydrogel showed similar trends, except in the region between 2800 cm^−1^ and 3000 cm^−1^, in which a peak at 2868 cm^−1^ was noted in the liposomal hydrogel spectrum due to the presence cholesterol. The two distinctive absorbance peaks at 1740 and 1591 cm^−1^ in the spectrum of chitosan (CS) represented the C=O bonding of –NHCO– (amide I) and the N–H bonding of the –NH2 (amide II) functional group, respectively. The FTIR spectrum of the lyophilized hydrogel without liposomes showed reduced absorption frequencies of amide I (1657 cm^−1^) and amide II (1556 cm^−1^). A similar trend was observed with regard to liposomal hydrogel, in which the intensities of the amide bands were 1568 cm^−1^ and 1411 cm^−1^, respectively. No new peaks could be observed in either hydrogel spectra. 

#### 2.1.4. Release Profile of VEGF from the Hydrogels

The cumulative release graph (Figure 4B) shows an initial burst release of 32.3 ± 3.5% of VEGF in the first 24 h in the hydrogel without liposomes group. Afterward, a fast release of 81.9 ± 3.8% of the total VEGF occurred over 21 days. On the other hand, in the first 24 h, the liposomal hydrogel group had a lower initial release of 13.5 ± 4.6%, followed by a sustained delivery of 31.0 ± 5.9%, resulting in a cumulative release of 44.5 ± 3.8% VEGF after three weeks. The findings confirmed the potential use of liposomal hydrogel in the sustained release of VEGF for an extended period of at least three weeks.

### 2.2. In-Vitro Characterization of Cell–Hydrogel 2.5D Culture

#### 2.2.1. Effect of Sustained Delivery of VEGF on Cell Morphology

**Figure 5:**
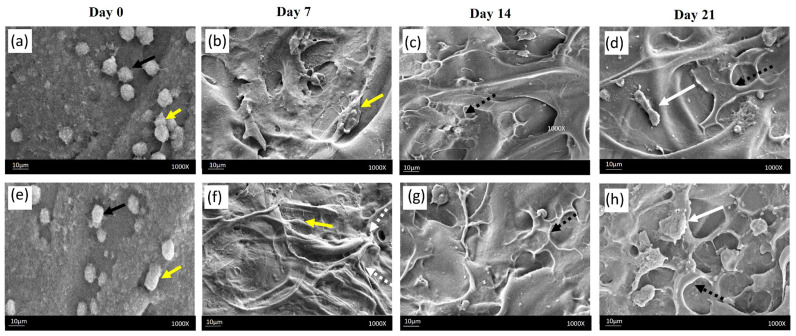
Surface cellular morphology viewed under FESEM at ×1000 magnification: Both spherical (black arrows) and spindle shaped cells (yellow arrows) were seen on day 0 (**a**,**e**). The presence of cytoplasmic extensions (white arrows, broken line) was observed on day 7 (**b**,**f**). Polygonal and flattened cells (black arrows, broken line) were seen on day 14 (**c**,**g**). On day 21, the liposomal hydrogel + VEGF group (**h**) showed denser cellular network and higher accumulation of mineralized nodules compared with the hydrogel without liposomes group (**d**). White arrows indicate deposition of calcium nodules.

The cellular morphology and interaction of t hydrogels were observed under FESEM on days 0, 7, 14, and 21 of culture. The FESEM images in Figure 5 demonstrate that cells with both spherical and spindle-shaped morphology were visible 24 h after seeding (day 0). On day 7, the cells displayed a spindle-like appearance, with cytoplasmic extensions contacting each other to form a network structure. On day 14, the cells became more polygonal and better spread, typical of differentiated osteoblastic phenotype. On day 21, calcium mineral nodules of varying sizes could be observed. Comparing the two hydrogel groups, it was revealed that the number of cells in both groups increased continuously from day 7 to day 21. The cells in the liposomal hydrogel + VEGF group spread more extensively, eventually covering the whole surface of the hydrogel, resulting in significantly higher deposits of apatite nodules by day 21 compared with the hydrogel without liposomes group, which exhibited more sparse mineral deposits. This result suggest that the liposomal hydrogel scaffold was most superior in the induction of cell differentiation and mineralization.

#### 2.2.2. Effect of Sustained Delivery of VEGF on Cell Viability

**Figure 6:**
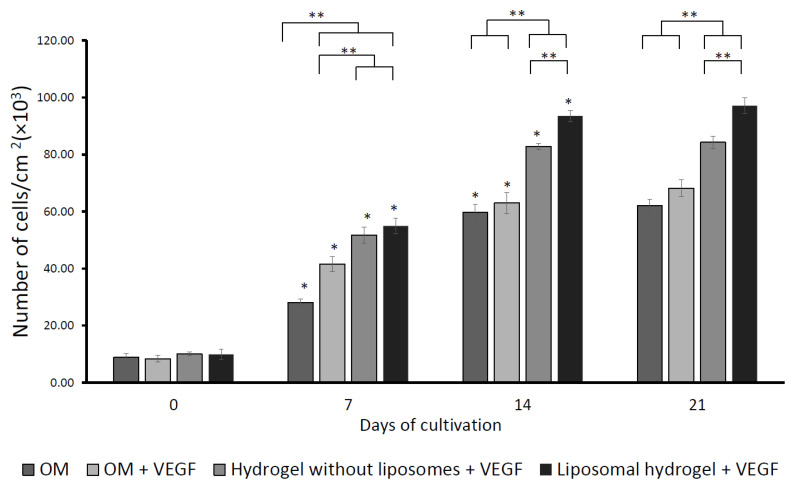
The effects of sustained delivery of VEGF on cell viability was determined with MTT assay on days 0, 7, 14, and 21 of cultivation. Each value was expressed as mean ± SD (*n* = 3 per group). * denotes statistical significance between different culture time points of each group (*p* < 0.05). ** denotes significant differences between groups at each time point (*p* < 0.05).

The impact of VEGF on cell viability using MTT assay is displayed in Figure 6. All the experimental groups demonstrated a time-dependent increment in the cell viability of MG-63 cells. On days 7, 14, and 21 of culture, the cell viabilities of the two hydrogel groups (hydrogel without liposomes and liposomal hydrogel) were significantly greater than the two control groups (OM and OM + VEGF) (*p* < 0.05). Between the control groups, the OM + VEGF group demonstrated significantly higher cell viability than the OM control group on day 7 of incubation (*p* < 0.05), due to the supplementation of VEGF as a bolus for one week. Nevertheless, from day 14 onwards, there were no significant differences between the control groups (*p* > 0.05). Between the two hydrogel groups, the cell viability in the liposomal hydrogel + VEGF group was significantly higher than the hydrogel without liposomes + VEGF group on days 14 and 21 of culture (*p* < 0.05). This finding indicated that the controlled delivery of VEGF via liposomal hydrogel significantly increased the cell viability of MG-63 cells over the 21-day culture period.

#### 2.2.3. Effect of Sustained Delivery of VEGF on ALP Activity

**Figure 7:**
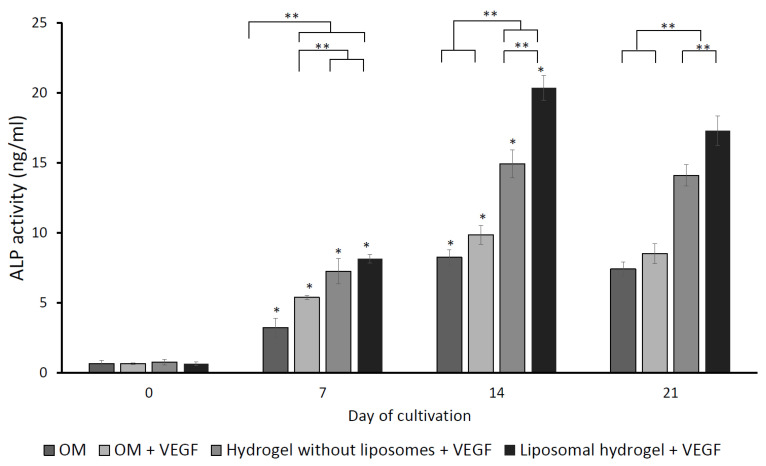
The effects of sustained delivery of VEGF on ALP activity assayed on day 0, 7, 14, and 21 of cultivation, and the ALP activity is expressed as nmol/ng. Each value is expressed as mean ± SD (*n* = 3 per group). * denotes statistical significance between different culture time points of each group (*p* < 0.05). ** denotes significant differences between groups at each time point (*p* < 0.05).

The effect of VEGF on ALP activity is displayed in Figure 7. The ALP activity of MG-63 cells in all the experimental groups climaxed on day 14 of cultivation but reduced on day 21. Between the control groups, on day 7 of incubation, the OM + VEGF group demonstrated significantly higher ALP activity than the OM-negative control group (*p* < 0.05). Nevertheless, no significant differences were noted between the control groups from day 14 onwards (*p* > 0.05). This is because VEGF was delivered as a bolus for one week. On day 7, 14, and 21 of culture, the ALP activities of the two hydrogel groups (hydrogel without liposomes and liposomal hydrogel) were significantly higher than the two control groups (OM and OM + VEGF) (*p* < 0.05). Between the two hydrogel groups, the ALP activity in the liposomal hydrogel + VEGF group was significantly greater than the hydrogel without liposomes + VEGF group on days 14 and 21 of culture (*p* < 0.05). This finding suggested that the sustained delivery of VEGF via liposomal hydrogel significantly enhanced the ALP activity of MG-63 cells over the 21-day experimental period.

#### 2.2.4. Effect of Sustained Delivery of VEGF on Matrix Mineralization

**Figure 8:**
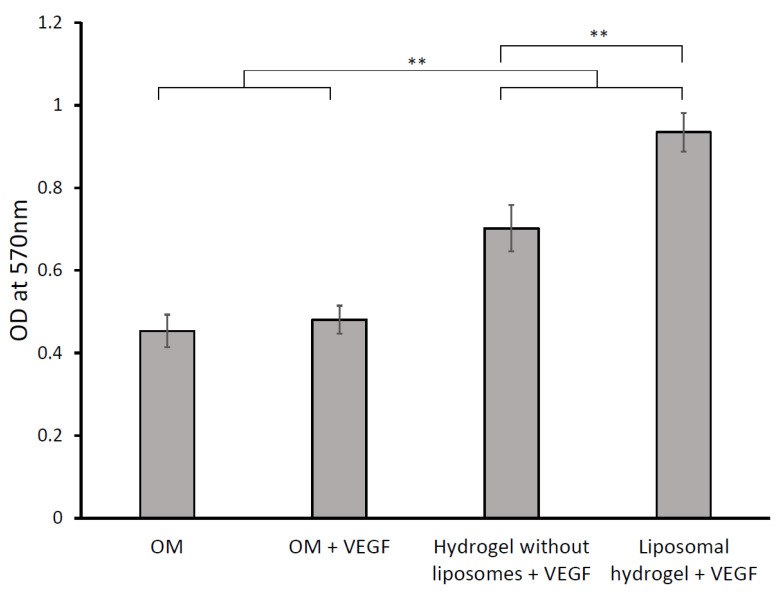
The effects of sustained delivery of VEGF on mineralization: Alizarin Red S staining was quantitatively assayed at 570 nm on day 21. Each value is expressed as mean ± SD (*n* = 3 per group). ** denotes significant differences between groups at each time point (*p* < 0.05).

In the later stages of the matured osteoblasts, calcium ions were incorporated into ECM, resulting in matrix mineralization. The calcium deposits formed were stained by Alizarin Red S after three weeks of culture and quantitatively assayed. The quantification results are shown in Figure 8. 

The calcium deposits in the cell-seeded liposomal hydrogel + VEGF group were the highest (*p* < 0.05). The mineral deposition in the hydrogel without liposomes + VEGF group was significantly greater than the control groups but lesser than the liposomal hydrogel + VEGF group (*p* < 0.05). The calcium deposition in the positive control group (OM + VEGF) was slightly greater than the negative control (OM), but the difference was not significant (*p* > 0.05). This result indicates that the sustained release of VEGF via liposomal hydrogel significantly enhanced the matrix mineralization of MG-63 cells over the three-week experimental period.

## 3. Discussions

The existing literature confirmed that 21 days of continued induction was necessary for the complete osteogenic process to take place [22]. Among the different carrier systems available, Ruel-Gariépy et al. (2000) [23] reported that preloading of bioactive molecules into microparticles or liposomes is necessary to achieve sustained delivery over more than one week from thermosensitive chitosan gels. The double encapsulation strategy using liposomal hydrogel in this study successfully fulfilled the requirement of sustained delivery of VEGF for at least three weeks. 

### 3.1. Characterization of Scaffold

The liposome formulation may influence the drug release profile. According to previous laboratory studies, the addition of cholesterol to liposome bilayers can prevent lipid exchange, thus providing an additional stabilizing effect [24]. In the current study, we followed an optimized formulation for the preparation of the liposomes, with dipalmitoylphosphatidylcholine (DPPC), dimethyldioctadecylammonium bromide (DDAB), and cholesterol (CH), at a molar ratio of 2:0.01:1 [25]. This formulation is in agreement with the proportions employed in most studies, i.e., 2:1 ratio (two parts of lipids and one part of cholesterol). However, the underlying reason for using these ratios is still unknown [24,26].

The parameters that determine the quality and suitability of a nanocarrier formulation for drug delivery include the average particle size/diameter, polydispersity index (PDI), and zeta-potential. The average particle size influences the drug release profile and bioavailability. This is because particle sizes on the order of 100 nm have a large surface-to-volume ratio, thus leading to a more controlled substance release [27,28]. The polydispersity index (PDI) measures the heterogeneity of size distribution of nanoparticles. PDI values smaller than 0.3 are deemed suitable for drug delivery purposes [29]. Zeta-potential measures the surface charge of nanoparticles in solution, and it is indicative of the colloidal stability. Zeta-potential values greater than +25 mV or less than −25 mV typically have a high level of stability [30]. Therefore, the VEGF-loaded liposomes with average particle size of 171 nm, PDI of 0.174, and zeta-potential of +38.5 mV in the current study were considered optimum for drug delivery. On average, the VEGF-loaded liposomes demonstrated a slight increase in particle size, PDI, and zeta-potential. However, the differences between liposomes with and without VEGF were statistically insignificant (*p* > 0.05). These findings showed that the loaded VEGF did not significantly alter the physical properties and morphology of the liposomes. 

The presence of liposomes within the polymeric chitosan hydrogel matrix did not disturb the gelation process. However, the gelation time for the hydrogel system was shortened after the liposomes were loaded. This was due to the alteration in the rheological properties of the hydrogel in the presence of secondary carriers (liposomes) [31]. The resultant liposomal hydrogel maintained the optimal microstructure (pore size and porosity), which was appropriate for cell attachment and growth. The average pore diameter of 100–325 μm was considered ideal for bone-tissue-engineered scaffolds according to Murphy et al. (2010) [32]. Previous studies found that bigger pore diameters (100–200 μm) favored bone regeneration after implantation, while smaller pore sizes below 100 μm resulted in fibrous tissue formation [33]. Porosity within the range of 80–90% was recommended for the cell penetration and vascularization of implanted scaffolds in bone defects [34]. Highly porous scaffolds (>90%) were associated with higher permeability and surface area for cell–biomaterial interactions. However, this resulted in inferior mechanical properties and higher degradability [35]. Therefore, the liposomal hydrogel with a pore diameter of 205.8 ± 25.5 μm and porosity of 84.0 ± 2.9% in this current study was acceptable for cell culture.

The FTIR analysis is a spectroscopic technique that uses infrared light to identify the chemical properties of scanned samples [36]. The FTIR result for hydrogel without liposomes from our study was similar to the study by Qin et al. (2018) [37]. The spectra of the lyophilized hydrogels with and without liposomes showed similar trends. This showed that the addition of liposomes did not alter the chemical structure of the hydrogel. Nevertheless, an absorbance peak at 2868 cm^−1^ was observed in the liposomal hydrogel spectrum, which corresponded to the tensile vibrations of C-H bonds and carbon cyclic rings. The region between 2800 cm^−1^ and 3000 cm^−1^ is a hallmark of the presence of cholesterol in liposomes [38]. Reduced wavenumbers of amide I and amide II bands as compared with the spectrum of chitosan were observed in both the hydrogel spectra. The FTIR results indicate that the sol–gel transition was due to the electrostatic interaction between negatively charged β-glycerophosphate and positively charged chitosan [39,40]. No new peaks could be observed in both hydrogel spectra, confirming the absence of chemical bonds between chitosan and β- glycerophosphate [39]. 

It was perceived that owing to the presence of liposome, the liposomal hydrogel had a lower initial release of about 13.5% in the first 24 h, with a cumulative release of 44.5% VEGF over 21 days, nearly half of that observed in the hydrogel without liposomes group. These results suggest that the presence of liposomes as secondary carriers provided a continuous release of VEGF below 40 ng/mL over 21 days, as required for the complete osteogenic process to take place. This result corroborates the findings of other studies, in which the sustained delivery of bioactive molecules was accomplished using liposomes [41,42]. Due to liposome leakage, VEGF first escaped from the liposomes into the hydrogel matrix. Later, VEGF was discharged into the culture medium, first through diffusion and then through degradation of the hydrogel matrix.

### 3.2. Effects of Liposomal Hydrogel on Cell Viability, Differentiation, and Mineralization

Osteogenesis comprises three major stages, proliferation, differentiation, and mineralization, taking place under continuous osteogenic induction for at least 21 days. Throughout these phases, expressions of specific osteogenic markers are temporally and sequentially organized [43,44]. Previous studies only compared the effects of VEGF-containing hydrogel without any secondary nanocarrier with negative controls over 14–21 days [45,46]. Therefore, in order to further investigate the effects of VEGF on osteogenesis in this study, three methods of delivery of VEGF, namely bolus delivery (OM + VEGF group), burst release (hydrogel without liposomes + VEGF), and sustained delivery (liposomal hydrogel + VEGF), were conducted, and the effects on cell proliferation, differentiation, and mineralization were evaluated for 21 days. 

The findings of the current study showed that the effect of sustained delivery of VEGF from liposomal hydrogel was the most superior compared with the hydrogel without liposomes carrier system reported by Wu et al. (2019) [45] and Elango (2023) [46], as well as the bolus delivery of VEGF without carrier [47]. This is explained by the sustained delivery of VEGF over the 21-day culture period. These results are in agreement with a previous study, in which the sustained delivery of bioactive molecules was accomplished using liposomes as secondary carriers [41]. 

The MG-63 cells exhibited favorable cell–material interaction on the hydrogels due to the presence of chitosan. The surface of chitosan promoted cell attachment by being positively charged, to which the negatively charged cell surfaces are naturally attached [48]. The porous and sponge-like microstructure of the hydrogels also facilitated cell attachment and proliferation [49]. While both hydrogels were proven to be non-cytotoxic and biocompatible, the liposomal hydrogel + VEGF was shown to be superior in the induction of cell differentiation and mineralization compared with hydrogel without liposomes + VEGF, as demonstrated by the more densely packed differentiated osteoblastic cells with a higher amount of calcium mineral deposits in the liposomal hydrogel + VEGF group.

VEGF stimulated the proliferative and differentiation potential of cells in a time-dependent manner, similar to other studies [47,50]. Increased proliferation and differentiation were due to the direct interaction of VEGF with the receptors expressed by the osteogenic cells, as proven by the presence of VEGFR-1 and VEGFR-2 receptors and VEGF binding proteins, Neuropilin-1 and -2 [51]. The study by Lee et al. (2012) showed that VEGF did not have a significant effect on the proliferation of human periodontal ligament stem cells, which was not in agreement with this study. However, the effects of VEGF were studied for a short period of time which did not extend beyond 5 days. In the current study, the proliferation stimulative effect of VEGF was no longer significant after the cell differentiation was initiated on around day 14. This current observation is consistent with the results in other studies also reporting that cell proliferation declines after two weeks of the osteogenic induction [46,52,53]. Both the hydrogels, with and without liposomes groups, exhibited higher ALP activities than the OM and OM + VEGF groups, consistent with the findings by Takagishi et al. (2006) [54], in which the promotion of osteogenic differentiation was reported in MG-63 cells cultured on gelatin hydrogels. The liposomal hydrogel + VEGF group expressed the highest ALP activity on day 14, because the osteoblastic differentiation was initiated in the MG-63 cells during this period, allowing the effects of persistent exposure of VEGF on osteoblastic differentiation to be fully expressed. 

Following the rise in ALP activity, matrix mineralization takes place. ALP breaks down pyrophosphate and inorganic phosphate, which results in the deposition of calcium minerals in the collagen fibers of ECM to form hydroxyapatite crystals from approximately the 14th day of culture [55]. Being a weak base, BGP was able to induce thermo-sensitive gelation when it was added to the acidic chitosan solution. At the same time, BGP also served as a source of organic phosphate for mineralization [56]. A previous study found that the sustained release of organophosphates from CS/BGP hydrogel may result in significantly greater calcium deposition [43]. Calcium mineral deposition exhibited the same trend as cell differentiation assays. The liposomal hydrogel + VEGF group induced the highest level of mineralization in MG-63 cell cultures. The current result again indicated that the synergistic effect between OM and sustained delivery of VEGF was the most effective in the induction of terminal differentiation and mineralization, supported by other studies conducted on human dental pulp stem cells [47,57]. 

Nevertheless, the optimal concentrations of VEGF for enhanced osteogenesis are controversial. The heterogeneity between studies may be influenced by the cell type, culturing conditions, and duration of time exposure of cells to VEGF. A recent study showed enhanced osteogenesis in human bone mesenchymal stem cells cultured for 21 days on a collagen–hydrogel scaffold [46]. However, the study by Aksel et al. (2017) on dental pulp stem cells showed that the highest levels of mineralization and expression of the osteogenic gene were seen when VEGF was given only during the first week of incubation, instead of continuous supplementation over 21 days [57]. Song et al. (2011) [58] reported that higher concentrations of VEGF over a longer incubation period could induce the upregulation of the inhibitor of DNA-binding 1 protein (Id1), which may retard terminal osteoblast differentiation and mineralization.

An in vitro study on human dental pulp stem cells demonstrated the highest level of mineralization when 40 ng/mL VEGF was used [47]. Another study showed enhanced osteogenesis of human periodontal ligament stem cells in vitro and in vivo in the presence of 25 ng/mL of VEGF [59]. The study by Wu et al. (2019) on dental pulp stem cells (DPSCs) reported that the odontogenic effect of 100 ng/mL VEGF loaded in a hydrogel delivery system was significantly greater than 100 ng/mL VEGF supplementation without carriers [45]. This finding was supported by the most recent study by Elango (2023), in which the differentiation and mineralization were accelerated when human bone mesenchymal stem cells were cultured on a collagen–hydrogel carrier system loaded with 100 ng/mL VEGF [46]. On the other hand, the studies by Behr et al. (2011) [60] and Aksel et al. (2017) [57] showed that different concentrations of VEGF (5–200 ng/mL) did not appear to induce significantly different osteoinductive effects on human adipose-derived stem cells and human dental pulp stem cells, respectively. Due to the varying results of the studies, until further confirmatory studies are conducted, the cumulative release of VEGF around 40 ng/mL over 3 weeks via chitosan hydrogel loaded with 100 ng/mL VEGF in the current study appears to be justified, because it would be safer to maintain the release of VEGF at a minimal level to avoid the undesirable side effects of excessive local concentrations of VEGF, such as hypertrophic bones and defective angiogenesis [61,62]. 

### 3.3. Limitations and Recommendations for Future Research

Although the MG-63 cell line is a useful cell model in bone research, it does not fully emulate the behavior of primary human osteoblast cells or induced osteoblasts from human pluripotent stem cells. For the determination of gelation time, rheological analysis using storage and loss modulus would be more accurate than the conventional tube inversion method. In addition, the parameters that are essential to maximize bone formation, such as the loading concentration and duration of delivery of VEGF, require further optimization. The 2.5D culture design could be upgraded to a 3D culture system with the usage of stem cells to further investigate the possibility of cell encapsulation combined with liposomal delivery of VEGF in bone regeneration. The release profile of VEGF from liposomes alone without hydrogel could be added in future studies for more thorough characterization of the release kinetics of VEGF.

## 4. Conclusions

Besides functioning as a scaffold in BTE, liposomal hydrogel is a very promising vehicle to deliver VEGF in a sustained manner in order to enhance the osteogenesis of osteoblast-like cells, as demonstrated by the increase in cell attachment, proliferation, differentiation, and mineralization. The liposome-loaded, 2% *w*/*v* chitosan, 6% *w*/*v* BGP hydrogel also displayed a unique thermosensitive profile for use in injectable form. The potential of this double encapsulation strategy in bone regeneration can be further investigated in preclinical in-vivo studies using human MSCs. 

## 5. Materials and Methods

### 5.1. Materials

VEGF165 Protein (Human Recombinant Animal Free) was obtained from Merck (Darmstadt, Germany). Chitosan (low molecular weight, 85% deacetylated) was acquired from Thermo Fisher Scientific (Waltham, MA, USA). Dipalmitoylphosphatidylcholine (DPPC), cholesterol (CH), dimethyldioctadecylammonium bromide (DDAB), beta-glycerophosphate (BGP), and other materials were bought from Sigma-Aldrich (Schnelldorf, Germany).

### 5.2. Preparation of Scaffold

#### 5.2.1. Preparation of VEGF-Loaded Liposomes

Liposome nanocarriers with encapsulated VEGF were fabricated using the thin lipid film hydration method [25]. DPPC, CH, and DDAB were mixed with chloroform at a weight ratio of 5 mg:1.31 mg:0.021 mg. Rotary evaporator was used to evaporate the chloroform at 55 °C under controlled low pressure until a dry lipid film was produced. Recombinant human VEGF165 was reconstituted in phosphate-buffered saline (VEGF/PBS). Liposomes with VEGF were produced by hydrating the thin lipid films with VEGF/PBS. After that, the sonication technique was used to produce liposomes in the form of small unilamellar vesicles. The liposome samples were kept in an ice-cold bath and sonicated using a probe sonicator with the following settings: 10 s ON, 10 s OFF intervals, 40% amplitude, and 750 W, for a total period of 15 min [63]. 

#### 5.2.2. Preparation of Thermoresponsive Hydrogels

An amount of 10 mL of hydrogel was prepared following the protocol mentioned in the study by Qin et al. [37]. Chitosan (CS) solution was made by mixing 200 mg CS powder in 8 mL acetic acid solution (0.75% *v*/*v*) at room temperature for 3 h. Subsequently, the dissolved CS was kept at 4 °C for 24 h to reduce the bubbles inside. BGP solution was prepared by adding 600 mg BGP in 2 mL distilled water and kept chilled at 4 °C. The chilled BGP was added drop by drop into the CS solution under continuous stirring in an ice bath for 10 min. The volume ratio of CS:BGP was 4:1. After that, liposomal hydrogel (liposomal hydrogel + VEGF) was obtained by adding the appropriate amount of VEGF-loaded liposomes into the CS/BGP mixture under gentle stirring for another 10 min to attain a final concentration of 100 ng/mL (*w*/*v*) VEGF [45,46]. Hydrogel without liposomes (hydrogel without liposomes + VEGF) was obtained by adding VEGF/PBS to attain the same final concentration of 100 ng/mL (*w*/*v*) VEGF. Gelation times for both hydrogels were ascertained using the tube inversion method in 37 °C water baths. The gelation point was established when there was no flow over 30 s, with the glass bottles being turned upside down [64].

### 5.3. Characterization of Scaffold

#### 5.3.1. Characterization of Physical Properties and Morphology of Liposomes

The particle sizes, polydispersity indexes (PDI), and zeta-potentials of liposomes (with and without VEGF) were ascertained in triplicates with the dynamic light scattering (DLS) method using a Zetasizer Nano ZS ZEN 3500 instrument (Malvern Instruments Ltd., Worcestershire, UK). The morphology of the liposomes with and without VEGF was observed by using a Talos-L120C transmission electron microscope (TEM, Thermo Scientific, Waltham, USA) at an accelerating voltage of 100 kV. The liposomes were stained with 1% ammonium molybdate (pH 7) on carbon-coated copper grids and analyzed under TEM. 

#### 5.3.2. Morphological Characterization of Hydrogel Using FESEM

After gelation, the hydrogels were lyophilized in a freeze drier overnight at −80 °C. After that, the samples were sectioned and analyzed under field emission scanning electron microscope (FESEM) (Merlin VP Compact, Zeiss, Oberkochen, Germany) to obtain the surface microstructure images of the hydrogels. The mean pore diameter of each sample was measured using the software Image-J by considering 20 pores [37].

#### 5.3.3. Porosity Determination of Hydrogels

The percentage of porosity was measured using the Archimedes method [65]. Initially, the dry weights of the lyophilized hydrogels (W_dry_) were measured and recorded. Then, the hydrogels were prewet with dehydrated alcohol and soaked in water. The submerged weights (W_sub_) were recorded when the hydrogels were submerged under water. After removal from the water, the weights were measured again (W_wet_). The porosities of the hydrogels were calculated with the formula below [65]:

Porosity (%) = [(W_wet_ − W_dry_)/(W_wet_ − W_sub_)] × 100%

where W_wet_ is the wet weight, W_dry_ is the dry weight, and W_sub_ represents the weight of the submerged hydrogel. 

#### 5.3.4. Characterization of Chemical Properties of Hydrogels Using FTIR Spectroscopy

Chemical properties of the lyophilized hydrogels and raw materials (CS and β-GP) were characterized using a SPECTRUM 400 FTIR spectrometer (Perkin Elmer, Waltham, USA) equipped with an attenuated total reflectance (ATR) accessory. All FTIR spectra (32 scans per spectrum) were collected within the wavelength range of 4000–500 cm^−1^ at a resolution of 4.0 cm^−1^ [37].

#### 5.3.5. Release Profile of VEGF from the Hydrogels

An amount of 2 mL of phosphate-buffered saline (PBS) was added to the hydrogels and incubated at 37 °C for 24 h. The leachate was collected and immediately frozen at −80 °C every 2 days over 3 weeks. After each leachate collection, the same volume of PBS was replenished. The concentrations of VEGF in the leachate were quantified utilizing the human VEGF enzyme-linked immunosorbent assay (ELISA) kit (FineTest, Wuhan, China). The cumulative release of VEGF (in ng/mL) was plotted as a function of time [45].

### 5.4. Cell–Hydrogel 2.5D Co-Culture

The cell line used for this study was the MG-63 cell line. The cell line was procured from the American Type Culture Collection (ATCC, Bethesda, MD, USA) (ATCC No. CRL-1427™). Complete medium (CM), containing Eagle’s minimum essential medium (EMEM), 10% (*v*/*v*) fetal bovine serum (FBS), and 1% (*v*/*v*) penicillin–streptomycin (Gibco, Grand Island, NY, USA), was used to culture the cells. Osteogenic medium (OM) composed of complete medium supplemented with 50 µg/mL L-ascorbic-2-phosphate (AAP) was used for osteogenic induction [66].

Hydrogels were prepared in aseptic environment according to the protocol mentioned previously. The sterile hydrogels were added into 24-well plates (500 µL/well). The hydrogels were incubated at 37 °C for 15 min for gelation to occur. MG-63 cells were seeded at 1 × 10^4^/cm^2^ seeding density on the hydrogel without liposomes + VEGF and liposomal hydrogel + VEGF scaffold groups and cultured in CM. After 24 h, the CM was discarded and replaced with OM. This marked day 0 of the analysis. In the positive control group, MG-63 cells were cultured without hydrogel in OM, initially supplemented with a bolus of 100 ng/mL (*w*/*v*) VEGF (OM + VEGF group). The VEGF was not replenished after one week to simulate the burst release effect. As a negative control, MG-63 cells were cultured without hydrogel in OM with no VEGF supplementation (OM group). The medium was replaced with fresh OM every 72 h. Subsequent experiments were performed in triplicates.

### 5.5. In Vitro Characterization of Cell–Hydrogel Co-Culture

#### 5.5.1. Cell Morphology Analysis Using FESEM

The cell attachment and morphology of the hydrogels were assessed using a field emission scanning electron microscope (FESEM) on day 7, 14, and 21. The samples were first fixed overnight in 2.5% *v*/*v* glutaraldehyde. Then, the samples were washed with PBS, followed by dehydration in a freeze drier for 24 h at −80 °C. After that, the samples were examined under FESEM (Merlin VP compact, Zeiss, Oberkochen, Germany) after sputter-coating.

#### 5.5.2. Cell Viability Analysis Using MTT Assay

MTT assay was used to investigate the cell viability on day 0, 7, 14, and 21. MTT solution and complete medium (volume ratio of 1:9) were added to each well and incubated at 37 °C for 4 h. Dimethyl sulfoxide (DMSO) in glycine buffer at pH 7.4 was used to dissolve the formazan crystal generated as a result of mitochondrial dehydrogenase activity. The solubilized formazan product was then transferred to a 96-well plate, and the optical density reading at 570 nm was recorded using an ELISA microplate reader (Varioskan Flash, Thermo Scientific, Waltham, USA). Each experiment was performed in triplicate. Number of cells/cm^2^ was plotted against the day of analysis.

#### 5.5.3. Cell Differentiation Analysis Using Alkaline Phosphatase (ALP) Assay

Alkaline phosphatase (ALP) is the key marker for determining osteoblastic phenotype. At the end of the 0-, 7-, 14-, and 21-day incubation time points, ALP activity was assayed using a Sensolyte^®^ pNPP alkaline phosphatase assay kit (AnaSpec, California, USA), as per the manufacturer’s protocol. In the presence of ALP, p-nitrophenol was released from p-nitrophenyl phosphate (PNPP) solutions. The concentration of p-nitrophenol was assayed in triplicate by recording the light absorbance at 405 nm using an ELISA reader (Varioskan Flash, Thermo Scientific, Waltham, USA). The result of the ALP assay was presented by plotting a graph of ALP activity (ng/mL) against the day of culture.

#### 5.5.4. Mineralization Analysis Using Alizarin Red S Staining Quantification

Alizarin Red S (ARS) staining was carried out to detect the calcium mineral deposit present. On day 21, the cell layers were washed with PBS, fixed with 10% formalin, and stained with 1% ARS (Sigma-Aldrich, Schnelldorf, Germany). Quantification of the calcium mineral deposition was performed by eluting the alizarin red stain with 10% (*w*/*v*) cetylpyridinium chloride. The absorbance was recorded at 570 nm using a microplate reader (Varioskan Flash, Thermo Scientific, Waltham, USA).

### 5.6. Statistical Analysis

The data analysis was conducted using the SPSS version 26. Descriptive data is expressed as mean ± standard deviation (SD). Normality of the data distribution was checked with Shapiro–Wilk test. An independent *t*-test was used for the comparison of microstructural characteristics between liposomes with and without VEGF. A Mann–Whitney U test was used for the comparison between hydrogel with and without liposomes. One-way analysis of variance (ANOVA) with the Bonferroni’s post hoc test was used to compare the differences between experimental groups in 2.5D co-culture. For the MTT and ALP results, one-way repeated measure ANOVA (Bonferroni post hoc) was used to compare the differences between experimental time points within the same group. A value of *p* < 0.05 was considered statistically significant.

## Figures and Tables

**Table 1:** Characterization of liposomes with and without VEGF. Data are expressed as mean ± standard deviation. (*n* = 8).

Formulation	Particle Sizes (d·nm)	PDI	Zeta-Potential (mV)	*p*-Value
Liposomes without VEGF	114 ± 28	0.221 ± 0.01	30.7 ± 12.8	*p* > 0.05
Liposomes with VEGF	171 ± 31	0.174 ± 0.03	38.5 ± 12.9	

**Table 2:** Average pore size and porosity of hydrogels with and without liposomes. Data represent mean ± standard deviation (*n* = 20).

Hydrogel Formulation	Pore Size (µm)	Porosity (%)	*p*-Value
Hydrogel without liposomes + VEGF	201.4 ± 24.5	81.1 ± 3.3	*p* > 0.05
Liposomal hydrogel + VEGF	205.8 ± 25.5	84.0 ± 2.9	

## Data Availability

The data presented in this study are available on request from the corresponding author.
